# Obstructive sleep apnea: a follow-up program in its relation to temporomandibular joint disorder, sleep bruxism and orofacial pain

**DOI:** 10.1186/s12903-023-03264-9

**Published:** 2023-08-19

**Authors:** Ruoyu Ning, Junjie Chen, Yanqin Lu, Jing Guo

**Affiliations:** 1 Department of Orthodontics, Xiangya Stomatological Hospital & Xiangya School of Stomatology, Hunan Clinical Research Center of Oral Major Diseases and Oral Health, Central South University, 72 Xiangya Road, Changsha, 410000, Hunan China; 2 Third Xiangya Hospital & Xiangya School of Medicine, Central South University, Changsha, Hunan China; 3 Engineering Laboratory for Biomaterials and Tissue Regeneration, Ningbo Stomatological Hospital, Zhejiang, China; 4 Savaid Stomatology School, Hangzhou Medical College, 435 Xinxing Road, Ningbo, 315000 Zhejiang China; 5 Department of Orthodontics, School and Hospital of Stomatology, Shandong University & Shandong Key Laboratory of Oral Tissue Regeneration & Shandong Engineering Laboratory for Dental Materials and Oral Tissue Regeneration, Cheeloo College of Medicine, Jinan, China

**Keywords:** OSA, TMJ, Tooth wear, Orofacial pain

## Abstract

**Objective:**

To evaluate the correlation between obstructive sleep apnea (OSA) and temporomandibular joint (TMJ) morphology, tooth wear condition, orofacial pain through a follow-up program.

**Materials and methods:**

Seventy one OSA patients were divided into three groups according to their (apnea hypopnea index) AHI: mild group (*n* = 23), moderate group (*n* = 24), and severe group (*n* = 24). All patients had OSA therapies around six months after confirm the diagnosis of OSA. The tooth wear score and orofacial pain condition of all patients were recorded via clinical examination. Cone beam computed tomography (CBCT) images were also taken when confirm the diagnosis of OSA (*T*_*0*_), 6 months after the diagnosis (*T*_*1*_), and 6 months after the OSA treatment (*T*_*2*_). Parameters indicating the condylar morphology and joint space were evaluated. The differences of clinical symptoms and TMJ conditions among *T*_*0*_*, T*_*1*_ and *T*_*2*_ time point were detected in the three groups respectively. The changes in *T*_*1*_*-T*_*0*_ and *T*_*2*_*-T*_*1*_ of all descriptions among three groups were also compared_*.*_ The correlations between AHI and clinical symptoms were detected with Spearman correlation analysis.

**Results:**

In mild group, there was no difference in all clinical symptoms and TMJ morphology among the three time points. Both in moderate and severe group, the condylar volume, superficial area, wear score, visual analogue scales (VAS), and R value (indicating condyle position) displayed significant differences among the three time points (*P* < 0.05). From *T*_*0*_ to* T*_*1*_, mild group displayed fewer decreases in the condylar volume and superficial area and fewer increases in wear score than that in moderate and severe group (*P* < 0.05). From *T*_*1*_ to* T*_*2*_*,* there was a greatest reduction in severe group for R value, and significant difference in the description of VAS and R value were found among the three groups. AHI was negatively correlated condylar volume and condylar superficial area, and was positively correlated with tooth wear score and VAS (*P* < 0.05).

**Conclusion:**

Moderate to severe OSA will aggravate orofacial pain and tooth wear, affect TMJ volume and superficial area, even change the location of condyles. Appropriate OSA therapies may be effective ways to alleviate these adverse effects in long-term.

## Introduction

Obstructive sleep apnea (OSA), affecting 9%—49% of the general population [1], is characterized by intermittent complete or partial collapse and obstruction of the upper airway during sleep, often accompanied by snoring, sleep structure disorders, nocturnal hypoxemia, daytime sleepiness and even apnea or hypopnea [2], which is associated with diverse comorbidities including hypertension, myocardial ischemia, stroke, arrhythmias, cognitive impairment, attention deficit and depression. Severe OSA even has an increased risk of all-cause mortality if left untreated in time [3, 4]. It has received more and more attention from respiratory medicine, otolaryngology, stomatology and other disciplines for its high morbidity and potentially fatal dangers [5]. A combination of factors accounts for the etiology and mechanism of OSA, including obesity, locally abnormal anatomy, impaired reflex mechanism of airway, unstable central respiratory regulation, and disordered neuromuscular function. Overnight full-channel PSG has been regarded as the gold standard for confirming and diagnosing OSA, allowing the quantitative recording of rhythmic masticatory muscular activity (RMMA), respiratory events, oxygen reduction events and other sleep parameters. Nasal CPAP is highly efficacious in keeping airway patency in OSA, as are oral appliances such as snore guard that hold the mandible in the forward position. Orthodontic and orthognathic treatment for OSA is also pretty vital, as the upper airway morphology is affected by craniofacial skeletal pattern [6]. For instance, an open bite accompanied by clockwise mandibular rotation influence the airway volume [7]; some congenital syndromes, eg. clefts, also have deformations within the upper airways and the nose itself [8, 9]. Under the circumstances, orthognathic surgery, mandibular advancement (MAD), rapid maxillary expansion (RME), et al. can significantly affect the upper airway morphology.

In addition to OSA, the disease sleep bruxism, orofacial pain and temporomandibular disorders (TMD) are also common in stomatological department [10, 11]. TMD is a collective term for a group of diseases referring pain conditions and functional jaw disabilities occurring, involving masticatory muscles, peripheral nervous system, and TMJs. Orofacial pain is the pain related with the hard and soft tissues of the head, face, and oral cavity [12], also including dental and TMD pain with significant influence on individual daily life, such as eating, drinking, and speaking [13]. Bruxism is a repetitive masticatory muscle activity characterized by clenching or grinding of the teeth and/or by bracing or thrusting of the mandible [14]. It is not a movement disorder or a sleep disorder in otherwise healthy individuals, but it can be a risk factor for certain clinical conditions or be a concomitant symptom of certain disease [15]. The pathogenesis of them has not been fully clarified and various therapies can be used for symptom relief.

Clinically, patients are usually diagnosed with two or more with above diseases, but clinicians sometimes take stopgap measures for them, such as one single elastic dental pad, physiotherapy, surgery, acupuncture, etc. In other word, such patients often fail to obtain standardized, comprehensive and complete treatment due to the single department they visited. There are problems in the outpatient clinic such as unclear treatment procedures and lack of standardized treatment system, which can only be solved through interdisciplinary diagnosis and sequence therapies.

Interestingly, these diseases in fact interact with one another and reinforce one another. According to a recent investigation, chronic TMD patients had markedly impaired sleep quality than healthy controls [16]. The prevalence of TMD signs and symptoms is significantly higher in untreated OSA patients when compared to controls [17]. A long-term cohort study also reported that OSA was a risk factor for the occurrence of first onset TMD [18]. Back in 2009, Smith et al [19] had emphasized the need to refer TMD patients complaining of sleep disturbance for polysomnographic evaluation; Additionally, it has been noted that patients with OSA are more likely to experience sleep bruxism [20, 21], and that the relationship between OSA and sleep bruxism depended on the degree of severity of OSA [22, 23]. The possible mechanism is that muscles contract and relax in a regular manner to keep the upper airway open. In the long run, euphoria-induced muscular traction will result in occlusal alterations and sleep bruxism [24], and this excessive bite force caused by bruxism may cause muscle fatigue, orofacial pain, and abnormal pressure on TMJ. Of course, there still are studies with different results. One polysomnographic research reported that the intensity of bruxism is associated to muscle activity, but not to pain related to TMD [25]. Wieckiewicz et al. also indicated that the distribution of TMD among sleep bruxers and non-bruxers is similar [26]. When talking about the relationship between OSA and orofacial pain, OSA has been associated with a higher risk of chronic pain [27], particularly with musculoskeletal pain disorders [28–30]. The patients with chronic orofacial pain proved to suffer from decreased sleep duration, increased stress and tiredness [31].

In order to carry out the interdisciplinary evaluation and comprehensive treatment for these diseases, understanding the association between OSA and TMD, bruxism, orofacial pain is very important, which would help provide references with new treatment options, would also help prevent and treat possible complications of OSA. However, limited study focused on the four diseases simultaneously, and such associations need further investigation and scientific evidence. The aim of this study is to explore whether OSA will affect sleep bruxism, orofacial pain and TMJ morphology, to explore if considerable OSA therapies have benefit in alleviating bruxism, orofacial pain and TMD symptoms, providing references for clinical practitioners. The null hypothesis is that no association would be found between OSA and TMD, bruxism, orofacial pain.

## Materials and methods

### Participants

This retrospective study included the data from 122 consecutive adults (77 male and 45 female) with OSA who received treatment from 2019 to 2023 at Ningbo Stomatological Hospital and Xiangya Stomatological Hospital, and the study was done at the Xiangya Stomatological Hospital of Central South University.

Subjects were selected according to the following criteria: (1) 25 to 45-year-old adults who complain of snoring or feel suffocated while sleeping, (2) meeting the diagnostic criteria for OSA [32] by polysomnography (PSG) with (apnea hypopnea index) AHI > 5. AHI represents the average number of apneas and hypopneas each hour during sleep, and the apnea or hypopnea must last at least 10 s or longer; along with typical clinical symptoms such as nocturnal respiratory symptoms, daytime excessive sleepiness, fatigue, growth retardation, hyperactivity, conduct disorder, and learning disabilities, (3) meeting the diagnostic criteria for TMD research (DC/TMD) [33], (4) having sleep bruxism in sleep lab and home, (5) permanent dentition, the number of existing teeth in the mouth > 20 (number of molar > 4). The exclusion criteria for this study were as follows: (1) trauma history, (2) TMJ surgery history and degenerative disease of temporomandibular joint [27](no specific osseous changes: subchondral cyst, erosion, generalized sclerosis, or osteophyte), (3) craniofacial anomalies, syndromes, severe asymmetries, clefts, craniosynostoses (Apert syndrome, Couson syndrome), or Treacher-Collins syndrome (4) OSA treatment history, ear-nose-throat (ENT) surgery history and TMJ surgery history, MAD treatment history, restorations in the oral cavity, (5) enamel hypoplasia due to dental fluorosis, tetracycline or other diseases, (6) long history of drinking carbonated beverages and other bad chewing habits, (7) cervical spine disorders and primary headache disorders, (8) history of gastroesophageal reflux disease (GERD) or use antipsychotic drugs.

**Fig.1:**
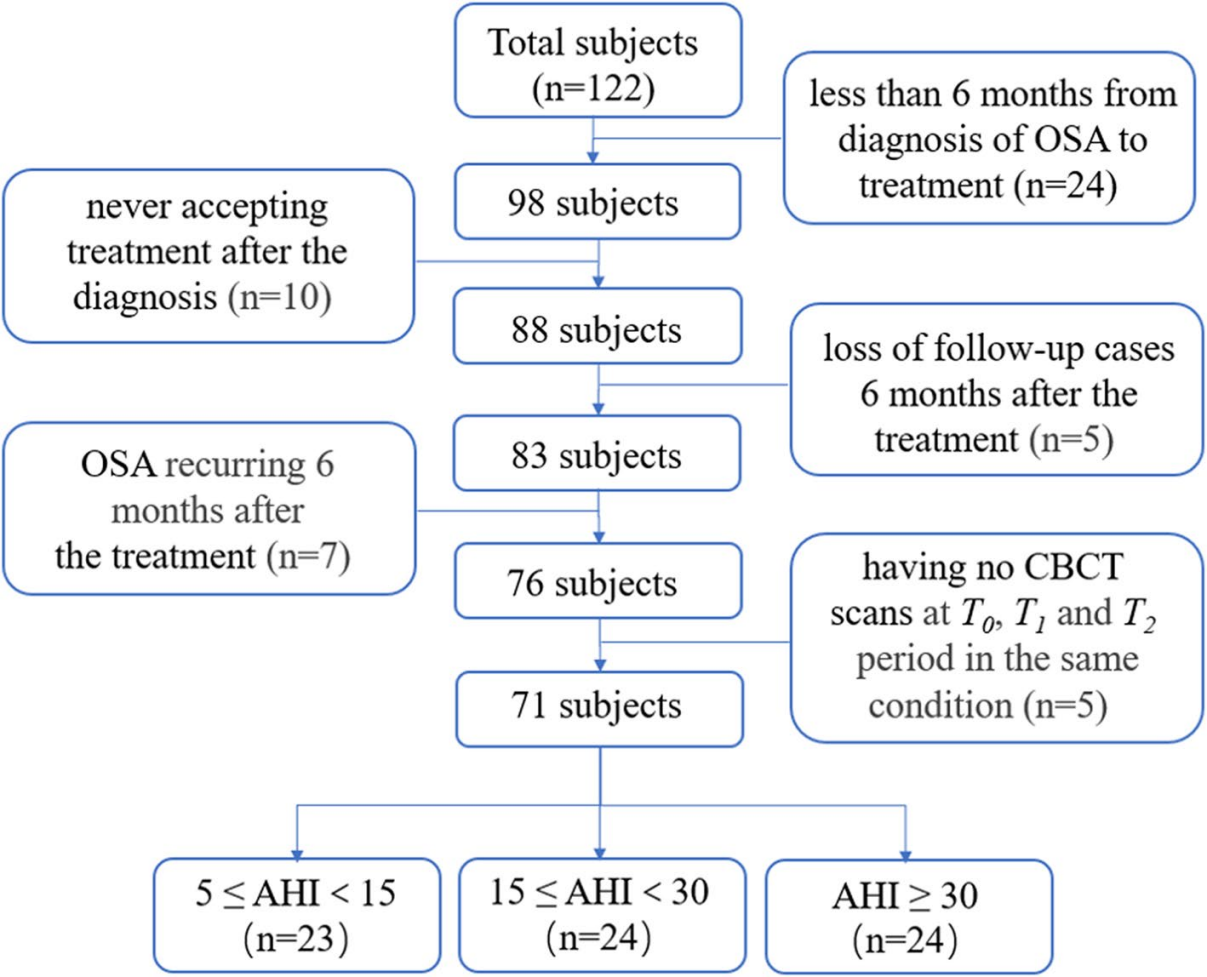
Study flow chart

Fifty-one subjects were excluded because of various reasons. 24 subjects treated their OSA less than 6 months from the diagnosis because of severe symptoms, the importance they attaching to the disease, and so on. 10 cases had not been treated since the diagnosis. 5 follow-up cases were lost 6 months after the OSA treatment. 7 cases relapsed 6 months after the treatment, and 5 subjects did not take CBCT scans when confirm the diagnosis of OSA, 6 months after the diagnosis, or 6 months after the OSA treatment under consistent shooting condition. Eventually, 71 subjects (aged 36.8 ± 3.5 years; 45 males and 26 females) who fulfilled the inclusion criteria were enrolled in the study (Fig. 1). The 71 subjects were divided into 3 groups according to the AHI value, diagnosed by PSG [34] in sleep lab, as follows: (1) mild group: *n* = 23, aged 35.8 ± 3.2 years, 5 ≤ AHI < 15, AHI mean value = 11.1 ± 2.0, (2) moderate group: *n* = 24, aged 36.5 ± 3.6 years, 15 ≤ AHI < 30, AHI mean value = 22.4 ± 4.9 (3) severe group: *n* = 24, aged 36.9 ± 3.3 years, AHI ≥ 30, AHI mean value = 52.8 ± 6.7.

The OSA therapies these subjects have received included nasal continuous positive airway pressure (CPAP) ventilation, loss weight (including professional weight loss guidance and/or liposuction), tonsillectomy and/or adenoidectomy, uvulo-palato-pharyngoplasty (UPPP), genioglossus advancement with hyoid suspension (GAHM), and orthognathic surgery. The type of OSA treatment chosen not only depended on the severity of OSA, but also need comprehensive consideration of patients' economic capacity, subjective wishes and tolerance to surgery. For instance, although CPAP is not standard procedure for mild OSA, it is one of treatment methods with quick return and low cost for the mild patients who had typical clinical symptoms. Specific treatment methods and efficiency of study groups are shown in Table 1.

**Table 1:** Basic information and polysomnographic parameter changes of the three study groups

Variables		mild group (*n* = 23)	Moderate group (*n* = 24)	Severe group (*n *= 24)	P
Gender (%)	Female	10(43.5)	9(37.5)	7(29.2)	*
	Male	13(56.5)	15(62.5)	17(71.8)	*
Age (year)	Range	28–40	25–41	28–43	
	Mean ± SD	35.8 ± 3.2	36.5 ± 3.6	36.9 ± 3.3	NS
BMI	Range	16.8–30.4	16.4–32.8	17.8–35.5	
	Mean ± SD	24.7 ± 3.5	27.0 ± 3.6	30.6 ± 3.7	*
Therapy (%)	CPAP	10(43.5)	11(45.8)	10(41.7)	
	Loss weight	17(73.9)	18(75.0)	18(75.0)	
	UPPP	2(8.7)	2(8.3)	2(8.3)	
	GAHM	1(4.3)	1(4.2)	1(4.2)	
	Orthognathic surgery	1(4.3)	1(4.2)	1(4.2)	
	Tonsillectomy and/or adenoidectomy	4(17.4)	5(20.8)	4(16.7)	NS
AHI	Before treatment	11.1 ± 2.0	22.4 ± 4.9	59.6 ± 7.0	*
	After treatment	1.2 ± 0.4	1.3 ± 0.5	4.7 ± 1.3	*
SpO_2_ (%)	Mean				
	Before treatment	92.7 ± 3.2	90.5 ± 3.6	86.7 ± 4.1	*
	After treatment	96.6 ± 4.0	95.2 ± 2.7	95.0 ± 3.9	NS
	Lowest				
	Before treatment	89.6 ± 4.9	82.3 ± 3.8	71 ± 5.6	*
	After treatment	94.5 ± 3.1	94.7 ± 2.9	93.6 ± 2.8	NS
ODI	Before treatment	8.1 ± 2.7	17.3 ± 4.0	52.5 ± 8.1	*
	After treatment	1.0 ± 0.2	1.1 ± 0.3	3.0 ± 1.2	*

^*^P < 0.05, SpO_2_: saturation of pulse oxygen, ODI: oxygen desaturation index

The clinical data including CBCT scans, intraoral slides, and visual analogue scales (VAS) were obtained at three different periods: confirm the diagnosis of OSA (*T*_*0*_), 6 months after the diagnosis (*T*_*1*_), and 6 months after the OSA treatment (*T*_*2*_). The subjects enrolled in our study didn’t receive the treatment within 6 months after confirm the diagnosis of OSA on account of neglect, busy job, procrastination, and expensive surgery, et al. Clinical data of 6 months after diagnosis and 6 months after OSA therapies were obtained through follow-up.

### TMD diagnosis and TMJ morphology

The diagnosis of TMD was based on DC/TMD, all subjects met the diagnostic criteria for TMD research (DC/TMD) [33]. Anterior disc displacement with reduction (ADDR) and anterior disk displacement without reduction (ADDWR) were diagnosed through magnetic resonance imaging (MRI). Limited opening and pain were ensured by measuring interincisor distance and palpation. All MRI data were calibrated and obtained by the same observer with patients not wearing fixed retainers, as retainers may blur and cause distortions to the image.

CBCTs of the three periods including *T*_*0*_, *T*_*1*_, and *T*_*2*_ of all subjects (acquired by New Tom 5G CBCT scanner, QR system, Verona, Italy, with exposure settings of 110 kV, 12-inch field of view, and 5.4-s exposure time) of all patients were accessed with the teeth in centric occlusion at the end of swallowing to make an exact diagnosis and acquire the information of the morphology of TMJs, which were calibrated and obtained by the same observer.

**Fig.2:**
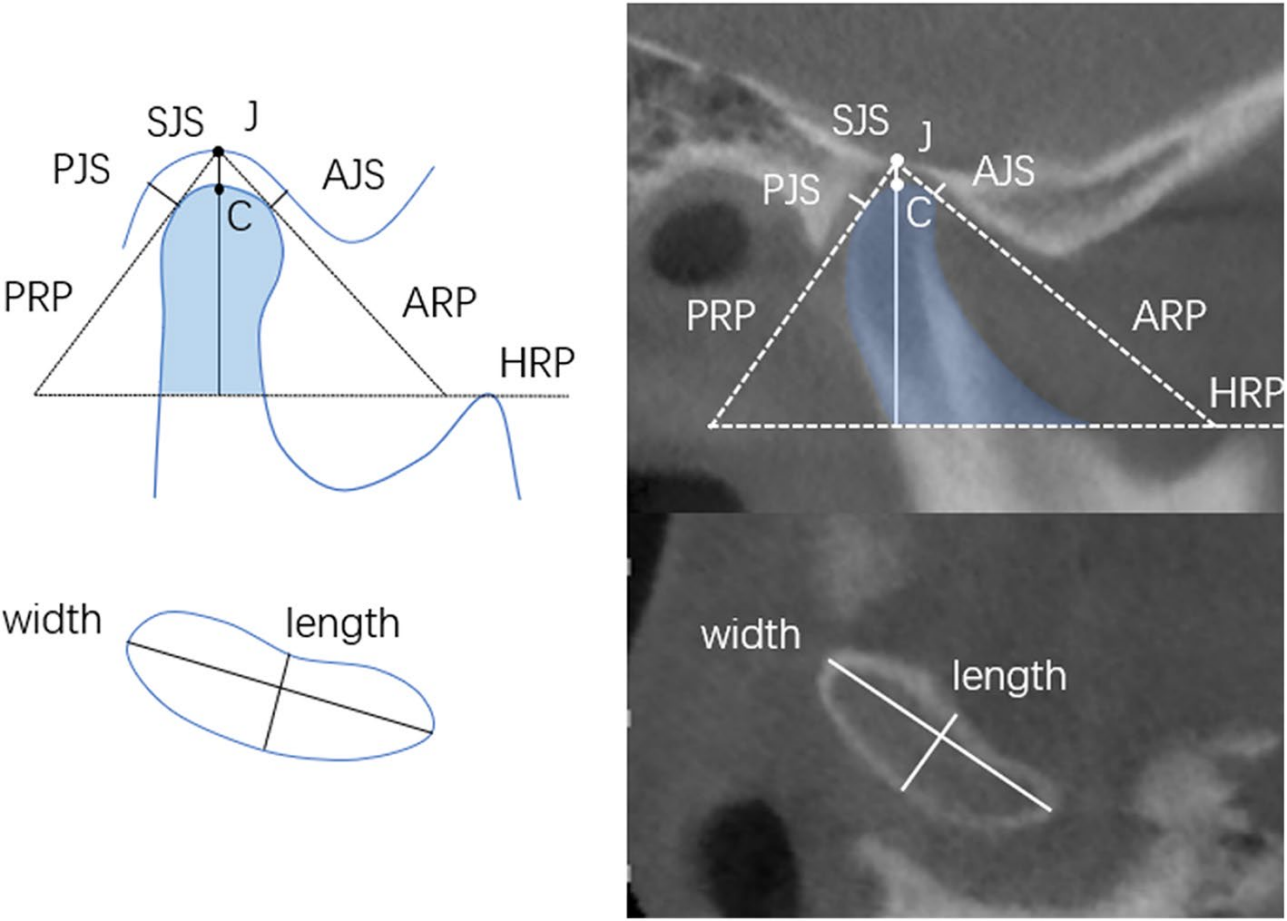
Illustration of the condylar morphology and join spaces

**Fig.3:**
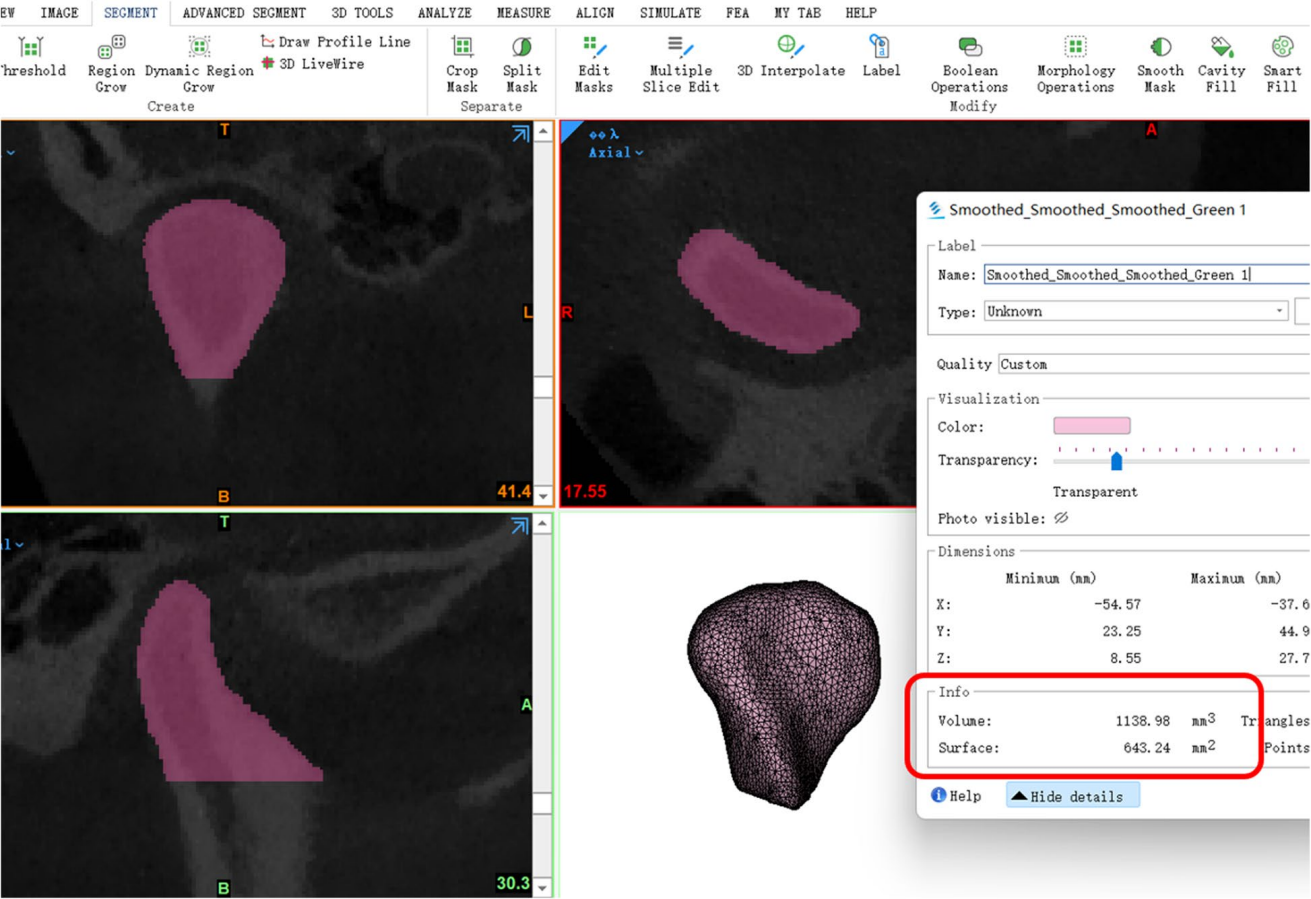
Measurement illustration of the condylar volume and superficial area in Mimics software

Descriptions on CBCTs were designed to measure the width, length, height, volume, and superficial area of condyles, as well as the joint space and position. All of the descriptions were measured in Mimics 21.0 software ((IBM, America). Head positions were standardized in CBCT according to the horizontal plane, positioned passing through the superior border of the external acoustic meatus and the inferior border of the infraorbital margin of both sides, and the sagittal plane, positioned passing through the anterior nasal spine, the internasal suture and the glabella. Detailed definition of condylar width, length, height, and joint space are shown in Table 2 and Fig. 2. 3D reconstruction of the target condyles was also performed in Mimics 21.0 software after the command Crop Mask, Region Grow, Split Mask, and Edit Mask, by setting three orthogonal sections: sagittal, coronal and cross-sections. The condylar volume and superficial area were acquired in the command Properties through executing the command Calculate Part (Fig. 3).

**Table 2:** Definition of measurements

Measurements		Definition
Reference point and plane	C point	the most superior point on condyle in sagittal view
	J point	the most superior point on TMJ fossa in sagittal view
	HRP	the horizontal reference plane: parallel to the lower border of the CBCT image and tangent to the most superior point of coronoid process
	ARP	the anterior reference plane: passing through J point and tangent to the leading edge of condyle
	PRP	the posterior reference plane: passing through J point and tangent to the trailing edge of condyle
Condyle	Width	maximum medial–lateral diameter of condyle in axis view
	Length	the anteroposterior diameter of condyle in axis view, defined by the line perpendicular to the Width line through midpoint
	Hight	the distance between C point and HRP in sagittal view
	Volume	volume of condyle above the interface HRP
	Superficial area	superficial area of condyle above the interface HRP
Joint space	SJS	superior joint space: the distance between point C and J
	AJS	anterior joint space: the distance between anterior TMJ fossa and the tangent point of ARP
	PJS	posterior joint space: the distance between posterior TMJ fossa and the tangent point of PRP
Condyle position	R value	R value = (PJS-AJS)/(PJS + AJS) *100% anterior condylar: R > 12%; posterior condylar: R < -12%; medium condylar: -12% < R < 12%

### Sleep bruxism diagnosis and tooth wear condition

Approaches for assessing bruxism in this study include non-instrumental and instrumental. Self- reported assessment of sleep bruxism continues is one primary tool in this research [11]. The patients who complained about grinding teeth when sleep were enrolled. Additionally, RMMAs were also recorded during PSG examination when meeting the following conditions: 1) At least 3 consecutive muscle activities for 0.25–2 s myoelectric burst; 2) Single myoelectric burst time is greater than 2 s; 3) each RMMAs event was preceded by at least 3 s of stable myoelectricity. However, RMMAs were recorded in only a subset of patients. Given the importance of self-reported assessment in the evaluation of sleep bruxism and the occasionality of a one-night PSG examination, we still included those who complained about bruxism but not found in sleep lab.

**Fig.4:**
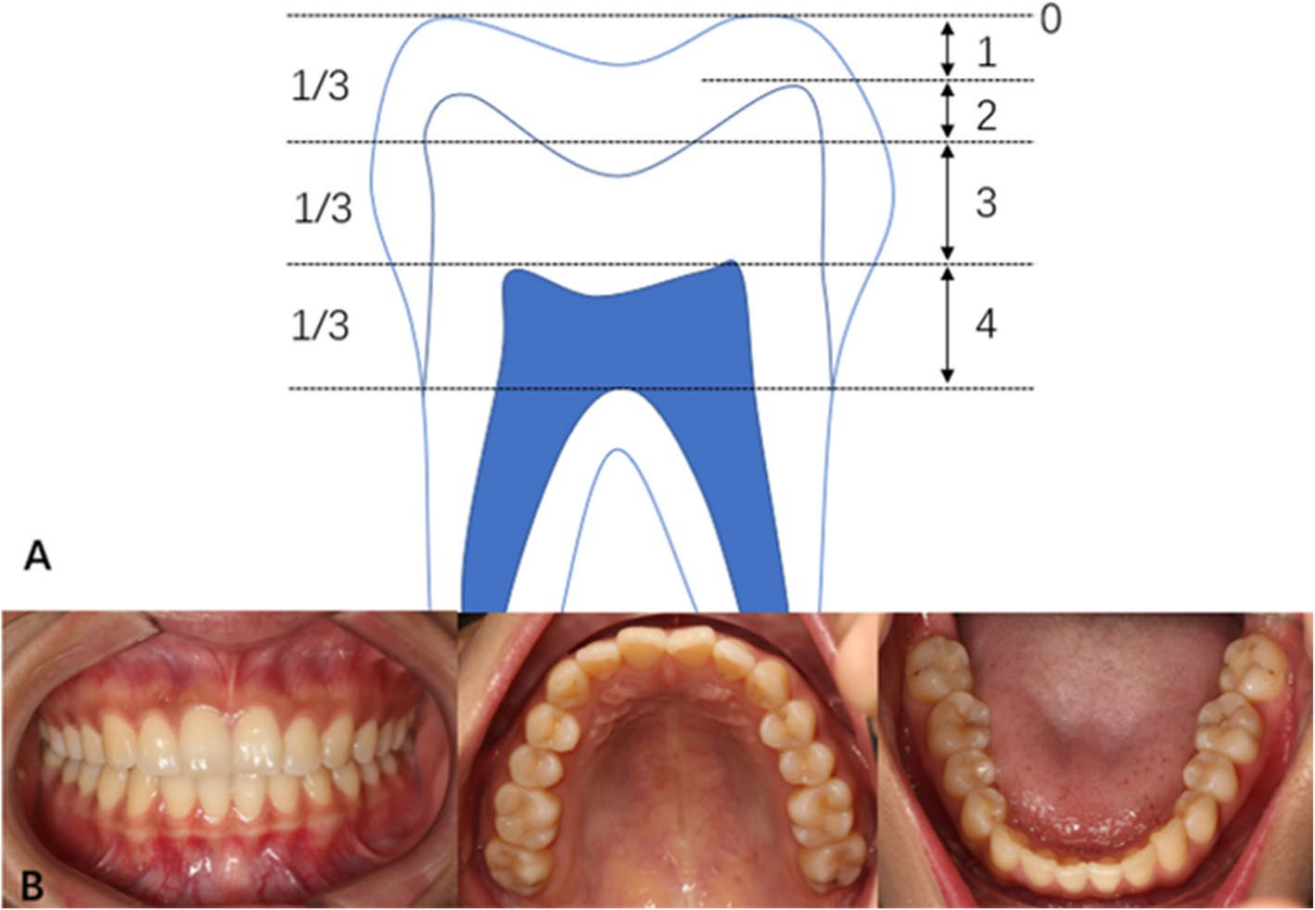
A: tooth wear scored with a 5-point ordinal scale reported by Lobbezoo; B: intraoral photographs of one subject (No. 27)

Tooth wear was scored with a 5-point ordinal scale reported by Lobbezoo [35]: 0 = no wear; 1 = visible wear within the enamel; 2 = visible wear with dentin exposure and loss of clinical crown height of ≤ 1⁄3; 3 = loss of crown height > 1⁄3 but < 2⁄3; and 4 = loss of crown height ≥ 2⁄3. Each subject was ultimately scored on the highest tooth wear level (Fig. 4).

To reduce random errors, serial numbers were assigned at random to each participant. All teeth of the first and third quadrants were examined in participants whose serial number was odd, while the second and fourth quadrants were examined in participants with an even serial number [36]. Wisdom teeth were excluded from the survey. For instance, the intraoral photographs of one subject (No. 27): the first and third quadrants were examined. The anterior teeth in light wear were recorded as 1, and the posterior teeth in serious wear was recorded as 2. The final score was 2 with the larger value (Fig. 4).

### Evaluation of orofacial pain and VAS

Approaches for orofacial pain in this study include patient’s pain complaint and clinical examination. The clinical tests include pain with opening jaw movements and palpation under the palpation pressure with 1 kg for 2 s. The examination included joint (arthralgia) and masticatory muscle (myalgia) palpation. The pain of temporalis, masseter, posterior mandibular region, submandibular region, lateral pterygoid area, and temporalis tendon were recorded, which replicated the patient's pain complaint [27].

**Fig.5:**
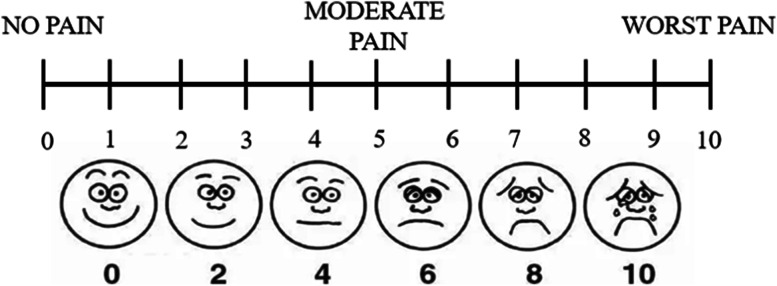
Visual analogue scales (VAS) for patients

Patient’s pain complaint was quantified by VAS, which was used for subjective evaluation of the degree of orofacial pain, including masticatory and joint area (0 = no pain, 10 = severe pain) (Fig. 5). The symptom of orofacial pain assessments at the three periods were done by one evaluator who was blinded to the history of each subject.

### Statistical analysis

All data were analyzed by IBM SPSS Statistics 21 software. One- and two-way analysis of variance (ANOVA) with Tukey tests was used to detect descriptions changes among *T*_*0*_, *T*_*1*_ and *T*_*2*_time points and among mild, moderate and severe groups. Correlations between AHI value and the clinical symptoms were assessed by Rho Spearman correlation analysis, and statistical significance was established at α = 0.05. To assess the reliability of these measurements, 30 subjects were randomly chosen. All measures were duplicated by the investigator R.N. An intraclass correlation coefficient (ICC) was used to determine the intra-observer reliability of the measurements through reliability analysis in SPSS. Reliability was divided into three categories: poor (ICC < 0.40), fair to good (0.40 ≤ ICC ≤ 0.75), and excellent (ICC > 0.75) [37].

## Results

The intra-observer reliability of the measurements of all descriptions using SPSS software was from fair to good to excellent, with ICC’s ranging from 0.673 to 0.916.

### Basic information and polysomnographic parameter changes (Table 1)

There were no significant differences in the age, type of OSA therapies and the interval of taking CBCTs among these three groups. However, gender and BMI of the study groups were significantly different. Severe group had the most percentage of male and fewest female, as well as the highest BMI index (30.6 ± 3.7). On the contrary, moderate group had the most percentage of female and fewest male, as well as the lowest BMI index (24.7 ± 3.5).

Before OSA treatment, AHI and ODI increased gradually from mild group to severe group(*P* < 0.05). After OSA treatment, the AHI values of all cases enrolled in this study were under 5, and the mean value were 1.2 ± 0.4 in mild group, 1.3 ± 0.5 in moderate group, 4.7 ± 1.3 in severe group. In addition, the ODI values were under 3 with the mean value of 1.0 ± 0.2 in mild group, 1.1 ± 0.3 in moderate group, 3.0 ± 1.2 in severe group. Before OSA treatment, both mean and lowest SpO2 decreased from mild group to severe group (*P* < 0.05). After OSA treatment, the three groups all had favourable mean and lowest SpO_2_.

### Proportion (%) of clinical symptoms before treatment (Table 3)

**Table 3:** Proportion (%) of clinical symptoms before treatment

Conditions		mild group (*n* = 23) No. of subjects (% within group)	Moderate group (*n* = 24) No. of subjects (% within group)	Severe group (*n* = 24) No. of subjects (% within group)
Orofacial pain	Myalgia	10(43.5)	13(54.2)	16(66.7)
	Arthralgia	2(8.7)	4(16.7)	5(20.8)
	Intra-auricular	2(8.7)	2(8.3)	2(8.3)
	Extra-auricular	1(4.3)	2(8.3)	3(12.5)
TMJ symptoms	Myofascial pain with limited opening	5(22.7)	4(16.7)	5(20.8)
	Disc displacement with reduction	14(60.8)	16(66.7)	18(75.0)
	Disc displacement without reduction	2(8.7)	2(8.3)	4(16.7)
	Disc displacement without reduction, with limited opening	0	1(4.2)	3(12.5)
Sleep bruxism	Sleep lab	10(43.5)	17(70.8)	16(66.7)
	Home	13(56.5)	7(29.2)	8(33.3)

43.5%, 54.2%, and 66.7% subjects in the mild, moderate, and severe groups had orofacial pain, respectively, most of which was myofascial pain. TMJ symptoms were examined according to RDC/TMD criteria The severe group had the highest proportion of disc displacement with reduction, up to 75.0%, and they were 60.8% and 66.7% in mild group and moderate group, respectively. The subjects in the mild group showed none of the subjects was diagnosed with disc displacement without reduction, with limited opening. Sleep bruxism in sleep lab refers to those grinded teeth in sleep lab and recorded by the PSG. Sleep bruxism at home refers to those complained about grinding teeth but not found in sleep lab. Moderate group had the most cases with sleep lab bruxism (70.8%) and the lowest cases with home bruxism (29.2%).

### Comparison of all descriptions among *T*_0_*T*_1_*T*_2_, in three groups respectively (Table 4)

**Table 4:** ANOVA and Tukey LSD analysis for clinical symptoms and TMJ morphology among different time points, in three groups respectively

Measurements		Mild group (*n *= 23)					Moderate group (*n* = 24)					Severe group (*n *= 24)				
		*T*_*0*_	*T*_*1*_		*T*_*2*_	*P*	*T*_*0*_	*T*_*1*_		*T*_*2*_	*P*	*T*_*0*_	*T*_*1*_		*T*_*2*_	*P*
Condyle	Width (mm)	19.2 ± 3.4	18.8 ± 2.8		18.9 ± 3.0	.466	18.5 ± 3.9	18.3 ± 2.5		18.3 ± 2.6	.190	17.7 ± 2.8	17.6 ± 2.5		17.7 ± 2.0	.093
	Length (mm)	8.5 ± 1.7	8.4 ± 1.9		8.4 ± 2.2	.372	7.8 ± 1.4	7.6 ± 1.8		7.7 ± 1.5	.214	7.3 ± 1.4	7.3 ± 1.8		7.1 ± 1.7	.149
	Hight (mm)	16.1 ± 2.0	15.8 ± 3.1		15.9 ± 2.6	.506	15.8 ± 2.2	15.6 ± 2.4		15.6 ± 1.9	.310	15.2 ± 2.6	14.9 ± 1.9		14.7 ± 2.4	.078
	Volume (mm^3^)	1409 ± 147	1402 ± 155		1400 ± 196	.651	1386 ± 102	1356 ± 95		1354 ± 86	.001*	1363 ± 108	1339 ± 101		1338 ± 87	.001*
	Superficial area (mm^2^)	723 ± 48	721 ± 52		721 ± 51	.865	693 ± 59	686 ± 62		684 ± 61	.001*	690 ± 62	679 ± 70		678 ± 65	.002*
Joint space	SJS (mm)	3.3 ± 0.8	3.4 ± 0.5		3.2 ± 0.3	.338	3.4 ± 0.2	3.6 ± 0.2		3.5 ± 0.7	.764	3.5 ± 0.4	3.6 ± 0.8		3.6 ± 0.7	.306
	AJS (mm)	2.2 ± 0.4	2.2 ± 0.2		2.1 ± 0.1	.264	2.2 ± 0.1	2.1 ± 0.1		2.0 ± 0.1	.585	2.4 ± 0.2	2.4 ± 0.1		2.2 ± 0.1	.275
	PJS (mm)	1.9 ± 0.1	1.8 ± 0.1		1.7 ± 0.2	.325	2.0 ± 0.1	1.9 ± 0.1		1.9 ± 0.1	.293	1.8 ± 0.1	1.7 ± 0.2		1.8 ± 0.2	.148
Condyle position	R value (%)	-10.9 ± 1.8	-11.0 ± 2.2		-9.6 ± 1.4	.107	-13.8 ± 1.0	-14.5 ± 2.0		-10.7 ± 0.4	.004*	-18.8 ± 2.8	-19.5 ± 3.2		-9.0 ± 1.4	.001*
Tooth wear	Wear score	1.9 ± 0.2	2.0 ± 0.1		2.0 ± 0.1	.562	2.2 ± 0.4	3.1 ± 0.3		3.0 ± 0.1	.008*	2.8 ± 0.1	3.2 ± 0.3		3.2 ± 0.7	.003*
TMJ area pain	VAS value	3.2 ± 0.7	3.2 ± 0.5		3.1 ± 0.4	.801	4.9 ± 1.3	5.6 ± 1.5		3.4 ± 0.9	.001*	6.3 ± 1.2	7.1 ± 1.9		3.5 ± 0.5	.001*
Measurement		Moderate group							Severe group							
		*T*_*0*_ * VS T*_*1*_		*T*_*1*_ * VS T*_*2*_			*T*_*0*_ * VS T*_*2*_		*T*_*0*_ * VS T*_*1*_			*T*_*1*_ * VS T*_*2*_		*T*_*0*_ * VS T*_*2*_		
Condyle	Volume	0.002*		0.588			0.001*		0.001*			0.891		0.001*		
	Superficial area	0.001*		0.654			0.001*		0.003*			0.483		0.002*		
Condyle position	R value	0.675		0.002*			0.003*		0.582			0.001*		0.001*		
Tooth wear	Wear score	0.003*		0.939			0.005*		0.004*			0.864		0.006*		
TMJ area pain	VAS value	0.006*		0.002*			0.001*		0.002*			0.001*		0.001*		

(*:*P* < 0.05)

In mild group, there was no difference in teeth wear, orofacial pain, and TMJ morphology among the three time points. The severe group had the largest wear score and VAS value, and smallest condylar volume and superficial area. Both in moderate and severe group, the ANOVA and Tukey LSD analysis indicated that condylar volume, superficial area, wear score, VAS value, and R value showed statistically significant differences among the three time points (Table 3). In both moderate and severe groups, condylar volume and superficial area showed a decreasing trend from *T*_*0*_ to* T*_*1*_ (moderate: *P* = 0.002, *P* = 0.001; severe: *P* = 0.001, *P* = 0.003), and no difference was found between *T*_*1*_ to* T*_*2*_*.* Wear score showed an increasing trend from *T*_*0*_ to* T*_*1*_ (*P* = 0.003, *P* = 0.004), and no difference was found between *T*_*1*_ to* T*_*2*_*.* VAS value showed an increasing trend from *T*_*0*_ to* T*_*1*_ (*P* = 0.006, *P* = 0.002), a decreasing trend *T*_*1*_ to* T*_*2*_ (*P* = 0.002, *P* = 0.001).

### Comparison of all descriptions among three groups in *T*_*1*_*-T*_*0*_ and *T*_*2*_*-T*_*1*_ (Table 5)

**Table 5:** Comparison of clinical symptom and TMJ morthopoly changes among three groups in *T*_*1*_*-T*_*0,*_* T*_*2*_*-T*_*1*_

Measurements		*T*_*1*_ *-T*_*0*_						*T*_*2*_ *-T*_*1*_					
		mild	moderate		severe		*P*	mild	moderate		severe		*P*
Condyle	Width (mm)	-0.4 ± 0.1	-0.3 ± 0.2		-0.3 ± 0.0		.374	0.1 ± 0.0	0.1 ± 0.0		0.1 ± 0.0		.953
	Length (mm)	-0.2 ± 0.0	-0.2 ± 0.1		-0.1 ± 0.0		.581	0.1 ± 0.1	0.1 ± 0.0		0.1 ± 0.0		.770
	Hight (mm)	-0.3 ± 0.1	-0.3 ± 0.1		-0.3 ± 0.2		.992	0.2 ± 0.1	0.1 ± 0.0		0.1 ± 0.1		.625
	Volume (mm^3^)	-7 ± 1.5	-31 ± 5.6		-25 ± 5.1		.001*	-1.9 ± 0.4	-2.0 ± 0.3		-1.8 ± 0.6		.131
	Superficial area (mm^2^)	-2 ± 0.8	-7 ± 1.5		-11 ± 2.7		.001*	-0.9 ± 0.2	-1.2 ± 0.5		-1.0 ± 0.3		.492
Joint space	SJS (mm)	0.1 ± 0.0	0.2 ± 0.1		0.1 ± 0.0		.258	-0.2 ± 0.1	-0.3 ± 0.1		-0.1 ± 0.0		.438
	AJS (mm)	0.1 ± 0.0	-0.1 ± 0.0		0.2 ± 0.0		.331	-0.1 ± 0.1	-0.1 ± 0.1		-0.2 ± 0.2		.651
	PJS (mm)	-0.1 ± 0.0	-0.1 ± 0.1		-0.2 ± 0.0		.547	-0.1 ± 0.0	0.1 ± 0.0		0.1 ± 0.1		.556
Condyle position	R value	-1 ± 0.2	-1.1 ± 0.2		-1.1 ± 0.3		.622	1.2 ± 0.4	3.6 ± 2.8		10.4 ± 3.3		.001*
Tooth wear	Wear score	0.1 ± 0.0	1.0 ± 0.3		0.9 ± 0.4		.007*	0.1 ± 0.0	-0.1 ± 0.0		0.1 ± 0.0		.974
TMJ area pain	VAS value	0.2 ± 0.1	0.7 ± 0.1		0.8 ± 0.2		.006*	-0.1 ± 0.0	-2.4 ± 0.7		-4.0 ± 1.6		.005*
Measurements		*T*_*1*_ *-T*_*0*_						*T*_*2*_ *-T*_*1*_					
		*P*						*P*					
		mild VS moderate		mild VS severe		moderate VS severe		mild VS moderate		mild VS severe		moderate VS severe	
Condyle	Volume	0.001*		0.003*		0.068		-		-		-	
	Superficial area	0.002*		0.001*		0.091		-		-		-	
Condyle position	R value	-		-		-		0.025*		0.001*		0.002*	
Tooth wear	Wear score	0.001*		0.001*		0.574		-		-		-	
Orofacial pain	VAS value	0.002*		0.002*		0.699		0.002*		0.001*		0.007*	

From *T*_*0*_ to* T*_*1*_*,* the amount of condylar volume and superficial area decreased in moderate and severe groups were significantly greater than that in mild group, and the amount of wear score and VAS value increased in moderate and severe groups were significantly greater than that in mild group. Besides there was no change for R value in three groups. From *T*_*1*_ to* T*_*2*_*,* R value increased in the moderate group (3.6 ± 2.8) and the severe group (10.4 ± 3.3), and significant difference in its changes was found between the mild group and the severe group (*P* = 0.001), between mild group and the moderate group(*P* = 0.025), as well as between the moderate group and the severe group (*P* = 0.002). Significant difference in the changes of VAS value was also observed among the three groups (*P* = 0.002, 0.001, 0.007).

### Spearman correlation results (Table 6, Fig. 6)

**Table 6:** Correlation between AHI value and the clinical symptoms

Measurements		Rho Spearman	*P*
Condyle	Width	-0.03	0.620
	Length	-0.01	0.791
	Hight	-0.02	0.688
	Volume	-0.34	0.013*
	Superficial area	-0.37	0.002*
Joint space	SJS	0.10	0.177
	AJS	0.09	0.254
	PJS	-0.08	0.339
Condyle position	R value	0.05	0.528
Tooth wear	Wear score	0.46	0.001*
Orofacial pain	VAS value	0.58	0.001*

**Fig.6:**
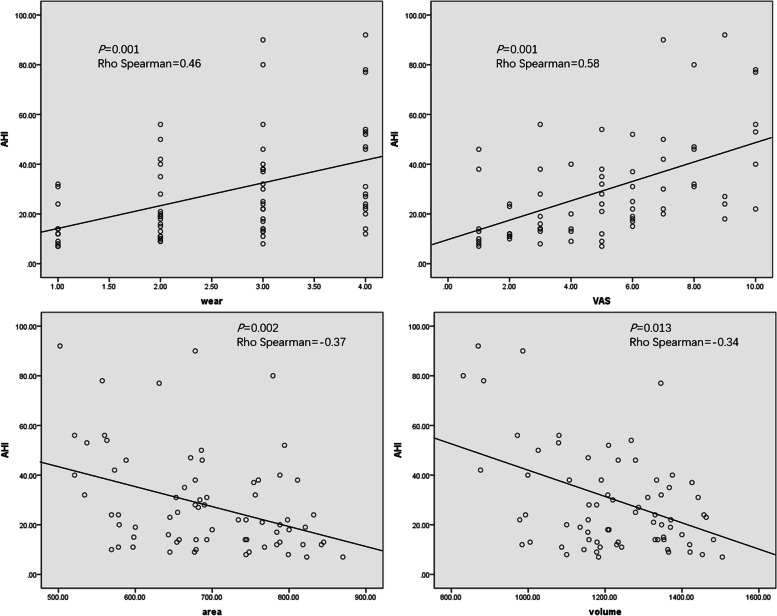
Scatter diagram of Spearman correlation analysis

Spearman correlation analysis displayed that AHI value was negatively correlated condylar volume and condylar superficial area (Rho Spearman = -0.34, -0.37; *P* = 0.013, 0.002). In addition, AHI value was positively correlated with tooth wear score and VAS value (Rho Spearman = 0.46, 0.58; *P* = 0.001, 0.001). The remaining descriptions was not statistically significant.

## Discussion

With the transformation of the traditional medical model to the bio-psycho-social medical model, the goal of oral disease treatment is to restore the morphology and function of the oromaxillofacial region to the best on the basis of multidisciplinary cooperation. Clinicians ought to provide patients with a suitable bite, to help them regain favorable chewing, swallowing, pronunciation and respiratory functions, while maintaining the continuous stability of the efficacy. OSA, TMD, bruxism, and orofacial pain are multidisciplinary and sometimes they may appear concomitantly without standardized, comprehensive and complete treatment. In order to explore comprehensive treatment for these diseases, understanding the association between OSA and TMD, bruxism, orofacial pain can help a lot, which would help provide references with new treatment options, or even prevent and treat possible complications of OSA.

The pathogenesis of sleep bruxism has not been fully clarified, which is related to emotional stress, anxiety disorders, OSA, consumption of certain groups of drugs, tobacco, alcohol, or coffee, and so on [38]. Long-term severe tooth grinding without treatment can lead to complications such as tooth sensitivity, periodontitis, dental fracture, and pulpitis. Sleep bruxism has been considered as a sign of a disorder in some healthy people, be regarded as a motor behavior with multifactorial etiology that can be a risk factor for a state of sub-health or certain diseases such as tooth wear, TMD, headaches, fatigue or pain in the masticatory muscles, and poor quality of sleep [39–41]. Recently, the association between OSA and sleep bruxism has drawn much attention, that is to say, OSA is considered as a new risk factor for sleep bruxism [42]. Currently, the treatment of sleep bruxism is limited to elastic dental pads to avoid extensive, continuous tooth wear at night. The result of this study indicated that considerable treatment of mild and moderate OSA may be beneficial in reducing sleep bruxism, as the wear score didn’t increase any more after OSA treatment. In the future, maybe clinicians can give other unconventionally potential treatment methods a try, especially when OSA and sleep bruxism associated together, such as biofeedback. Gender and BMI of our study groups were significantly different, but it is now generally accepted that there notably no differences between female and male, and just prevalence decreases with increasing age [43]. The association between BMI and bruxism is also negligible. So, we can conclude the difference may have no influence on the results.

One of the hypotheses connecting sleep bruxism and OSA is that bruxism at night plays a protective role against OSA by protruding the jaw and restoring upper airway patency [44, 45]. The mandibular depressors such as genioglossus will occur rhythmic movements, and the subsequent RMMA resulting in mandibular protrusion and opening of the airways [46]. On the other hand, oromaxillary muscles in the state of euphoria traction may lead to occlusal changes and become one of the causative factors of bruxism [20]. So, bruxism may be a secondary manifestation with OSA, that is to say OSA is a risk factor for sleep bruxism. However, Sjöholm [47] reported this mechanism was not adequate to prevent the airway from collapsing in too severe OSA, as individuals with severe OSA have significantly less deep sleep. That is inconsistent with the results of our study, and we suspected the race factor might explain the discrepancy, which need to be justified by the divergent pattern of sleep structure in patients with different degrees of severity of OSA.

Chronic widespread pain with psychologic factors, increasing age, and female gender are risk factors for chronic orofacial pain [48, 49]. Orofacial pain is in connection with high morbidity, negative social effects, and high medical expense if not accurately diagnosed and managed. People coming to the clinic often complain about headache, neck pain, vertigo, tinnitus, phonophobia, photophobia, strange taste, or bruxism [50]. Sometimes there is no good treatment other than massage and physical therapy. Our results showed that no treatment of moderate and severe OSA may aggravate myofascial pain and arthralgia. The significant reduction of VAS value six months after reasonable OSA therapies also indicated OSA treatment sometimes can relieve orofacial pain although no statistical significance was observed in the mild group. In addition, Spearman correlation analysis displayed that AHI value was positively correlated with VAS value. We assume that the following reasons may account for such phenomenon.

A famous hypotheses connecting sleep pain and OSA is that sleep deprivation and recurring nocturnal hypoxemia in OSA individuals during sleep would enhance pain sensitivity, promote inflammation, and increase spontaneous pain [51–53], would lead to myalgia, tenderness, and chronic fatigue. Choy [54] reported that sleep deprivation and fragmented sleep impairs descending pain-inhibition pathways that are crucial for controlling and dealing with pain. Besides long-term tooth grinding may cause fatigue, tenderness, and tension pain in the masticatory muscles.

Main symptoms of TMD are pain, joint friction, irregular or limited mandibular function. The current mainstream non-surgical therapies of TMD include hard occlusal splint therapy, counselling, exercises, massage, manual therapy, and so on. Psychosocial factors, immunity, occupational strain, TMJ overload and its anatomical factors are recognized as important risk factors of TMD among adults [55]. Furthermore, certain previous studies also have elaborated the relationships between OSA and TMD [16, 17, 56–58]. Alessandri et al. reported that the prevalence of TMD signs and symptoms is significantly high in untreated OSA patients [11], which is consistent with our study. Our results indicated that no treatment of moderate and severe OSA may lead to a decrease in the condylar volume and superficial area. After OSA treatment, the decrease of condylar volume and superficial area in moderate and severe group stopped along with a decreased R value. In addition, Spearman correlation analysis displayed that AHI value was negatively correlated condylar volume and superficial area. It can be considered to provide new references for TMD therapies based on this association. In other word, OSA treatment may be effective in reducing the morphological changes of TMJ.

Gender plays a controversial role in the occurrence and development of TMD. Although it is now generally accepted that TMD and orofacial pain are more common in female, several researches were inconsistent with such consensus. Razi et al. reported there was no significant relationship between condyle morphology and gender [59]. Görürgöz et al. thought being male was associated with radiographically detectable degenerative findings in the mandibular condyle [60]. In our study, gender has statistical differences in the three study groups. Contrary to that consensus, severe group had a more absorbed condyle and higher VAS, but the group also contains more male. From this, we hypothesized the gender difference didn't affect our results.

We analyze that bruxism may be related in TMD occurrence and development. It is possible that in people with OSA, apnea and hypopnea resulted from the airway collapses and obstructs will produce an abnormal muscle environment and varying degrees of neuromuscular dysfunction. TMJ is subjected to abnormal stress and pulling force that is different from that of normal people under such neuromuscular dysfunction. By this time the fragile condyle, condylar neck, and joint disc may shift, finally leading to TMJ injury. Diatchenko [61] indicated that people with OSA have an increased stimulation of the sympathetic nervous system, that underlies an incremental risk of developing first-onset TMD [62].

There are certain limitations of this follow-up analysis. Not every subject has been recorded with RMMAs during PSG examination, and no other bruxism instrumental method was performed. Only self-reported assessment was used in the study is a limitation; Gender and BMI have statistic differences in the three groups. Although we believe the impact of this point is small enough, whether the results dependent on gender and BMI will be investigated in our next study. However, despite causality couldn’t be inferred from observational data alone, the findings from this research could imply that OSA contributes to the onset of sleep bruxism and orofacial pain to some extent, which could still provide important clinical references.

### Clinical applications

OSA, orofacial pain, sleep bruxism and TMD has become more common and problematic among the general individuals. The complexity of pathogenesis and anatomy at the orofacial region contributes to challenging diagnosis, differential diagnosis and treatment for clinicians. A better understanding of underlying physiological mechanisms on the associations among OSA, orofacial pain, sleep bruxism and TMD may help clinicians do differential diagnosis and provide them with new options of therapies.

As there is no consensus on the treatment protocol for TMD, bruxism, and orofacial pain with OSA, it has not been definitively guided which disease should be treated first when two or more of these symptoms occur at the same time. In this context, clinicians need to consider bruxism, orofacial pain and TMD as clinical predictors for OSA, and ought to consider not only symptoms of bruxism, TMD and orofacial pain, but also OSA. Because OSA may influence mandibular function and cause the presence of secondary orofacial pain, bruxism and TMD. For example, clinicians can implement a sleep quality investigation for the patient to confirm if there is any secondary symptom, and the treatment of OSA may relief the clinical consequences of bruxism, orofacial pain or TMD if so. This strategy is not an overall solution to all situation, but a series of comprehensive one. In our opinion, it is more systematic than the specific but limited therapy.

For a better management of patients with OSA, chronic orofacial pain, TMD or bruxism, comprehensive understanding of the correlative mechanism among these different conditions would be helpful. Interdisciplinary treatment including physicians, dentists and otolaryngologists is also very essential.

## Conclusion

Moderate to severe OSA is a risk factor for orofacial pain, sleep bruxism and condylar absorption, which affect condylar volume and superficial area. Appropriate OSA therapies may be effective ways to relieve orofacial pain, reduce tooth grinding at night, and prevent condylar absorption in the long run.

## Data Availability

The data that support the findings of this study are available from Ningbo Stomatological Hospital and Xiangya Stomatological Hospital but restrictions apply to the availability of these data, which were used under license for the current study, and so are not publicly available. Data are however available from the authors upon (Ruoyu Ning, nrysdu@163.com) reasonable request and with permission of Ningbo Stomatological Hospital and Xiangya Stomatological Hospital.

## Abbreviations

OSA: Obstructive sleep apnea
PSG: Polysomnography
VAS: Visual analogue scales
AHI: Apnea hypopnea index
TMD: Temporomandibular disorder
TMJ: Temporomandibular joint
RMMA: Rhythmic masticatory muscular activity
CPAP: Continuous positive airway pressure
ANOVA: One- and two-way analysis of variance
